# Treating posttraumatic stress disorder remotely with cognitive therapy for PTSD

**DOI:** 10.1080/20008198.2020.1785818

**Published:** 2020-07-14

**Authors:** Jennifer Wild, Emma Warnock-Parkes, Hannah Murray, Alice Kerr, Graham Thew, Nick Grey, David M. Clark, Anke Ehlers

**Affiliations:** a Department of Experimental Psychology, University of Oxford, Oxford, UK; b Oxford Health NHS Foundation Trust, Oxford, UK; c Department of Psychology, King’s College London, London, UK; d Sussex Partnership NHS Foundation Trust, Worthing, UK

**Keywords:** PTSD, cognitive therapy, cognitive behaviour therapy, trauma-focused, COVID-19, remote therapy, TEPT, terapia cognitiva, terapia cognitivo conductual, centrada en el trauma, COVID-19, terapia remota, PTSD, 认知疗法, 认知行为疗法, 聚焦创伤, 新冠肺炎, 远程治疗

## Abstract

Delivering trauma-focused cognitive behavioural therapy to patients with PTSD during the COVID-19 pandemic poses challenges. The therapist cannot meet with the patient in person to guide them through trauma-focused work and other treatment components, and patients are restricted in carrying out treatment-related activities and behavioural experiments that involve contact with other people. Whilst online trauma-focused CBT treatments for PTSD have been developed, which overcome some of these barriers in that they can be delivered remotely, they are not yet routinely available in clinical services in countries, such as the UK. Cognitive therapy for PTSD (CT-PTSD) is a trauma-focused cognitive behavioural therapy that is acceptable to patients, leads to high rates of recovery and is recommended as a first-line treatment for the disorder by international clinical practice guidelines. Here we describe how to deliver CT-PTSD remotely so that patients presenting with PTSD during the COVID-19 pandemic can still benefit from this evidence-based treatment.

## Abstract (es)

Brindar terapia cognitivo conductual (TCC) centrada en el trauma a pacientes con Trastorno de Estrés Postraumático (TEPT) durante la pandemia de COVID-19 plantea desafíos. El terapeuta no puede reunirse con el paciente en persona para guiarlo a través del trabajo centrado en el trauma y apoyarlo con otros componentes del tratamiento; y por otra parte, los pacientes tienen restricciones para llevar a cabo las actividades relacionadas con el tratamiento y los experimentos de comportamiento que impliquen contacto con otras personas. Si bien se han desarrollado tratamientos de TCC centrados en el trauma en línea para el TEPT, que superan algunas de estas barreras en el sentido de que pueden administrarse de forma remota, todavía no están disponibles de forma rutinaria en los servicios clínicos de los países, como en el Reino Unido. La terapia cognitiva para el TEPT (TC-TEPT) es una terapia cognitiva conductual centrada en el trauma que es aceptable para los pacientes, conduce a altas tasas de recuperación y se recomienda como tratamiento de primera línea para el trastorno según las pautas internacionales de práctica clínica (APA, 2017; International Society of Traumatic Stress Studies, 2019; National Institute of Health and Clinical Excellence, 2018). Aquí describimos cómo administrar TC-TEPT de forma remota para que los pacientes que se presentan con TEPT durante la pandemia de COVID-19 aún puedan beneficiarse de este tratamiento basado en la evidencia.

## Abstract (zh)

在COVID-19疫情期间向PTSD患者提供针聚焦创伤的认知行为疗法是一个挑战。治疗师无法亲自与患者会面, 以指导他们完成聚焦创伤的工作和其他治疗措施, 并且患者在开展治疗相关的活动和涉及与他人接触的行为实验方面受到限制。尽管已为PTSD开发了聚焦创伤的在线CBT治疗, 因其可以远程提供治疗克服了一部分上述障碍, 但在英国等国家的临床服务中仍无法常规使用。 PTSD认知疗法(CT-PTSD)是一种聚焦创伤的认知行为疗法, 患者可以接受, 可达高康复率, 并被国际临床实践指南推荐为该疾病的一线治疗方法(APA, 2017; International Society of Traumatic Stress Studies, 2019; National Institute of Health and Clinical Excellence, 2018)。在此, 我们描述如何远程提供CT-PTSD, 以使得COVID-19疫情期间的PTSD患者仍可从这种循证治疗中受益。

## 1. Introduction

Delivering trauma-focused cognitive behavioural therapy (CBT) to patients with post-traumatic stress disorder (PTSD) during the COVID-19 pandemic poses challenges. Imposed restrictions to reduce the spread of the virus mean that therapists are unable to meet with patients in person and must deliver their therapy remotely over video conferencing or the telephone. Patients are also restricted in carrying out treatment-related assignments that could involve contact with other people due to social distancing requirements. These restrictions raise questions about how therapists can deliver trauma-focused CBT safely and effectively. Technological advances have made remote therapy delivery possible (Olff et al., 2019). Whilst online CBT treatments for PTSD (e.g. Knaevelsrud & Maercker, 2007; Lange, Van De Ven, & Schrieken, 2003; Litz, Engel, Bryant, & Papa, 2007; for meta-analyses see Kuester, Niemeyer, & Knaevelsrud, 2016; Lewis, Roberts, Bethell, Robertson, & Bisson, 2018) offer a potential solution in that they can be delivered remotely, are acceptable to patients (Simon et al., 2019), and include trauma-focused work, they tend to have moderately high drop-out rates in clinical trials (means 22% to 25.8% in the meta-analyses of Kuester et al., 2016; Lewis et al., 2018) and are not yet routinely available in clinical services in countries, such as the UK. Apps are available to manage PTSD symptoms, yet most have not been evaluated in randomised controlled trials. The exceptions are PTSD Coach, which has demonstrated efficacy (Kuhn et al., 2017; Sander et al., 2020), whilst SUPPORT Coach has not (van der Meer, Bakker, Zuiden, Lok, & Olff, 2020). CBT for PTSD delivered over the telephone achieved significant reductions in PTSD symptomatology in one clinical trial (DuHamel et al., 2010), and treatment via video-conferencing has been found to be acceptable to patients (Ashwick, Turgose & Murphy, 2019). Here we describe how to deliver remotely (over video conferencing or by telephone) one of the recommended and effective CBT treatments for PTSD: trauma-focused cognitive therapy for PTSD (CT-PTSD).

CT-PTSD is based on Ehlers and Clark (2000) cognitive model of PTSD and is recommended as a first-line treatment in international clinical guidelines (APA, 2017; International Society of Traumatic Stress Studies, 2019; National Institute of Health and Clinical Excellence, 2018). Several randomised controlled trials have demonstrated that face-to-face CT-PTSD is effective and acceptable to patients who have experienced a wide range of traumatic events, with low drop-out rates (3% on average in randomised trials) and high patient satisfaction scores in adults (Ehlers, Clark, Hackmann, McManus, & Fennell, 2005; Ehlers et al., 2003, 2014, 2020b) and children and adolescents (Meiser-Stedman et al., 2017; Smith et al., 2007). In these trials, CT-PTSD led to large improvements in PTSD symptoms, disability, depression, anxiety, and quality of life, and over 70% of patients (intent-to-treat) recovered from PTSD, i.e. did not meet diagnostic criteria any longer. CT-PTSD was shown to be superior to supportive therapy (Ehlers et al., 2014) and self-help (Ehlers et al., 2003). Three effectiveness studies (Duffy, Gillespie, & Clark, 2007; Ehlers et al., 2020a, 2013) implemented CT-PTSD in routine clinical services with a wide range of patients including those with serious social problems, severe comorbidity, multiple traumatic events and losses who were treated by both experienced and trainee therapists and showed good outcomes. The results support CT-PTSD as an evidenced-based treatment for PTSD following a wide range of traumas.

**Figure 1.**
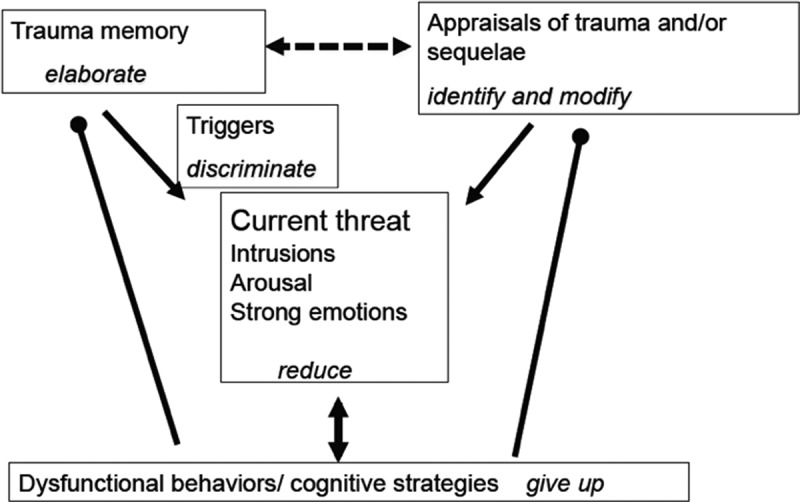
Ehlers and Clark (2000) cognitive model of PTSD with treatment goals (reprinted with permission from Ehlers, 2013).

CT-PTSD is guided by Ehlers and Clark (2000) cognitive model for PTSD, shown in Figure 1. This model proposes that PTSD is characterised by a sense of serious current threat, which has two sources: the nature of the trauma memory (which leads to intrusions of the trauma and other re-experiencing symptoms) and excessively negative appraisals of the trauma and/or its sequelae. Cognitive and behavioural strategies intended to control the sense of threat maintain the problem, such as safety behaviours, memory suppression and rumination.

CT-PTSD has three aims: (1) to elaborate and update the trauma memory in order to reduce re-experiencing symptoms, (2) to modify negative appraisals and (3) to change strategies that maintain the patient’s sense of threat whilst at the same time helping the patient to reclaim/rebuild activities in their life that provide a sense of worth and meaning. CT-PTSD is typically delivered in twelve sessions (90 min for sessions that include memory work) with three optional booster sessions spaced one month apart. Core interventions are:

- An *individualized case formulation*, which serves as the framework for therapy. Treatment procedures are tailored to the formulation,

*Reclaiming/rebuilding your life assignments*,

- *Updating trauma memories*, a three-step procedure designed to link less threatening meanings to the worst moments in memory,

*Discrimination training with triggers of re-experiencing*,

- A *site visit,*
- *Changing problematic appraisals* of the traumas and their sequelae, which is closely integrated with the *updating memories procedure*,
- *Dropping unhelpful behaviours and cognitive processes*, including *behavioural experiments,*
- A *blueprint to* summarizes what the client has learned in treatment.

Below we describe these core interventions and how to adapt them for remote working. We start with considering some challenges of working remotely and suggestions of how to address them.

### 1. 1. Engaging patients safely in remote trauma-focused therapy

As in face-to-face therapy, a good therapeutic relationship is important for supporting patients to feel safe to engage in their treatment and with their trauma memories. Evidence suggests that a strong therapeutic alliance can be achieved in remote therapies for PTSD (e.g. Knaevelsrud & Maercker, 2007). The collaborative therapeutic style of cognitive therapy helps the patient build trust and maintain a sense of control over the chosen steps in therapy throughout. It is important for the therapist to explicitly convey the message that they are with the patient, supporting them through the therapy, and to be empathic about all aspects of the patient’s behaviour, thoughts and feelings. While in face-to-face therapy the therapist’s nonverbal behaviour (e.g. nodding, handing tissues) and facial expressions can amplify supportive utterances, this possibility is more restricted in remote therapy, especially in phone calls. Therapists will need to rely more on verbal statements and a warm tone of voice to express their support and empathy. Therapists should regularly ask for the patient’s feedback about the content and pace of the interventions (‘Does this make sense?’, ‘What do you think would be a way for us to test …’), looking for doubts and ‘blocking thoughts’ that need to be addressed as they may impede engagement. Sharing screen in videoconferencing can help working collaboratively and make sure there is a joint understanding between patient and therapist. For therapy conducted via the telephone, sharing documents and media files via email or SMS can have the same function if the patient consents.

The therapist needs to foster a safe therapy environment, ensuring that patients have privacy during their sessions and problem-solve with them if this is not possible to achieve at home (e.g. they may speak from the car while parked in a quiet space). It is important for therapists to be working in a private place where they will not be overheard, and to use headphones for telephone and video calls. If they are conducting the session over video conferencing facility, they may reassure the patient that they have complete privacy by allowing their camera to pan their room to show that they are alone.

The therapist may wish to email or send a photo of themselves to patients if they are conducting the therapy over the telephone to help to increase the sense of safety and establish a relationship. It will be important for the therapist to address any concerns the patient has about working remotely. This may include sharing the advantages of remote working, such as the patient being in their own environment whilst completing the therapy and the opportunity to work together with trauma triggers that are in the patient’s home. Early success experiences are likely to facilitate engagement with the treatment. As in face-to-face delivery, from the first session onwards, cognitive therapy encourages experiential exercises and assignments that are likely to increase hope and provide early shifts in emotion and intrusive memories, such as the reclaiming/rebuilding life assignments, early updating of trauma memories and trigger discrimination techniques described below. It will also likely be valuable to involve supportive friends and family where possible, particularly with reclaiming life activities, behavioural experiments and trauma site visits.

Engagement with treatment can also be supported and facilitated using regular text messaging with the patient, with their agreement. The therapist is advised to follow their service’s guidance on messaging patients. This is usually done from the therapist’s work mobile phone or via an online text messaging programme. This can be for general encouragement, specific reminders for therapy tasks, and eliciting feedback.

One of the tasks of the therapist in trauma-focused treatments is to help the patient engage with trauma memories sufficiently so that the associated threatening meanings can be changed, but not to over-engage with the memories so that they lose awareness of the present situation or become so emotionally aroused that they cannot process new conclusions/meanings from therapy. Remote working poses challenges in that the therapist is less able to see the nuances of facial expressions linked to emotion. When working on the telephone, other signs of emotional engagement such as gestures or body postures from the trauma are also absent. A thorough assessment of dissociative tendency and risk of self-harm is therefore especially important before remote trauma-focused interventions commence. For remote sessions, we ask patients to identify reminders of their safe present circumstances (the ‘here and now’) and have them with them during the sessions. These are objects that can be used to help the patient focus on what is different between the time of the trauma and the safe present day. Examples include a strong scented lip balm, a strong tasting sweet or a photo taken with a significant other since the trauma. The therapist’s voice is an important cue that was not present at the time of the trauma and can also help to keep the patient’s attention in the here and now. Taking verbal ratings of ‘nowness’ (i.e. the extent to which the memories appear to happen in the here and now rather than in the past) and distress when working with trauma memories ensures that the work being carried out is meaningful and effective, and is perhaps even more important than in face-to-face treatment.

In terms of managing clinical risk, the therapist is advised to follow their service’s safeguarding protocol. This process should include a clinical assessment at the outset with a screen for suicide risk and risk to others to determine suitability for PTSD treatment over intervention to address risk. Many patients with PTSD experience comorbid depression and may experience suicidal ideation before or during the course of treatment. It is important to monitor symptom levels session-by-session, including the presence and/or change in suicidal ideation (Luxton et al., 2014). This can be monitored with the Patient Health Questionnaire (PHQ; Kroenke, Spitzer, & Williams, 2001), paying close attention to item 9. The therapist may collaboratively develop a plan of how the patient may stay safe in a crisis, and what steps may be taken if the therapist is concerned for their safety.

### 1. 2. Individualised case formulation

Whether CT-PTSD is delivered in person or remotely, the therapist will conduct an assessment of current problem(s), agree the patient’s goals for therapy, and develop a case formulation which guides treatment and may be further developed as therapy progresses. At the core of the formulation is the patient’s experience of current threat (i.e. threat to safety or self-worth), and the therapist will help identify the processes that maintain this. This will include features of the trauma memory, such as its ‘here and now’ quality and disjointedness. Often the worst moments are disjointed from information in memory that could reduce the sense of threat and are re-experienced as intrusions. The therapist will help the patient identify cues that trigger intrusions and other re-experiencing symptoms, and the patient’s cognitive and behavioural strategies for dealing with them, such as suppression of intrusive memories, rumination, and dissociation. The therapist will also draw initial attention to unhelpful meanings linked to the trauma and its consequences (e.g. ‘I attract danger’ ‘I am a bad person’ ‘People will look down on me if they knew how I reacted’ ‘People cannot be trusted’), which threaten the patient’s sense of self and safety.

Session by session administration of symptom measures, such as the PTSD Checklist for DSM-5 (PCL; Weathers et al., 2013) or the revised Impact of Event Scale (Weiss, 2007) is recommended as it enables therapist and patient to see what interventions are working and to make adjustments to therapy, as appropriate. Remote completion of questionnaires has been demonstrated to be acceptable to patients and feasible (Price et al., 2018). We also strongly recommend the use of process measures that assess the key psychological processes targeted in therapy. They are given throughout treatment to monitor progress and plan sessions along with symptom measures. Four commonly used process measures are described here in Table 1.

**Table 1.** Commonly used process measures in CT-PTSD.

Process Measure	Purpose
*Post-traumatic Cognitions Inventory* (PTCI; Foa, Ehlers, Clark, Tolin, & Orsillo, 1999) or a shortened version that can be given weekly (e.g. Kleim et al., 2013).	To assess trauma-related appraisals linked to feelings of overgeneralised fear, anger, guilt, shame, permanent change, alienation or persistent degradation.
*Responses to Intrusions Questionnaire* (RIQ; e.g. Clohessy & Ehlers, 1999).	To assess unhelpful responses to intrusive memories, such as rumination, suppression and numbing.
*Safety Behaviours Questionnaire* (SBQ; e.g. Dunmore, Clark, & Ehlers, 2001).	To assess excessive precautions the patient may take to minimise perceived risk.
*Unwanted Memories Questionnaire* (e.g. Michael, Ehlers, Halligan, & Clark, 2005).	To assess qualities of intrusive memories that contribute to the sense of current threat such as their “nowness”.

These measures are available on the Oxford Centre for Anxiety Disorders and Trauma (OxCADAT) resources website: www.oxcadatresources.com and can be sent to the patient to complete before each session, for example, by email.

For an example of the diagram of the full cognitive model as it relates to PTSD associated with a stay in an intensive care unit, please see Murray et al. (2020). The full model helps the therapist to consider all relevant processes but is not typically shared with patients in the early stages of therapy. Instead, therapist and patient draw out sub-components of the model (relevant maintenance cycles) as they are introduced into treatment and used as the rationale for specific interventions. When working remotely by videoconferencing, the therapist can open a blank document and share their screen with the patient in order to draw a maintenance cycle collaboratively. This can then be emailed or sent via messaging to the patient if they consent. If the therapy is being conducted on the telephone, the therapist can draw out the maintenance cycle and send it to the patient during or after the session (see example below).

A common maintenance cycle drawn out early on with patients relates to the suppression of unwanted memories. An experiential exercise to investigate the impact of thought suppression may be conducted with patients on the phone or by video conferencing as in face-to-face therapy. Patients are asked to think about whatever they want for a minute, anything at all, except a pink bunny rabbit sitting on the therapist’s shoulder (or some other memorable image). After a minute, the patient is asked what happened and they typically report that the pink bunny rabbit popped into their mind even though they tried to push such image out. The therapist may then guide the patient to discover that the effects of suppressing images is similar to suppressing memories. They pop back. She/he may include the effects of suppression and other strategies the patient uses in response to unwanted memories in a maintenance cycle and share screen or email to the patient.

For example, Kaja’s father was living in a care home. He developed breathing problems during the COVID-19 pandemic and paramedics were called to help him. When they arrived they told her that he had a choice: to die in the care home with his family close to him or to die in hospital with no family. He became very ill over the next few days. On one visit, he could not sit up, walk or speak and his breathing was very laboured. Kaja thought he would die. That day, they got the results of his COVID-19 test and it was negative. The local GP treated him for a chest infection and he recovered. However, Kaja developed PTSD. She had images of seeing him struggling to breathe with the feeling that he would die at any moment. When these images popped into her mind, she pushed them away or dwelled about why the paramedics did not try to help him.

**Figure 2.**
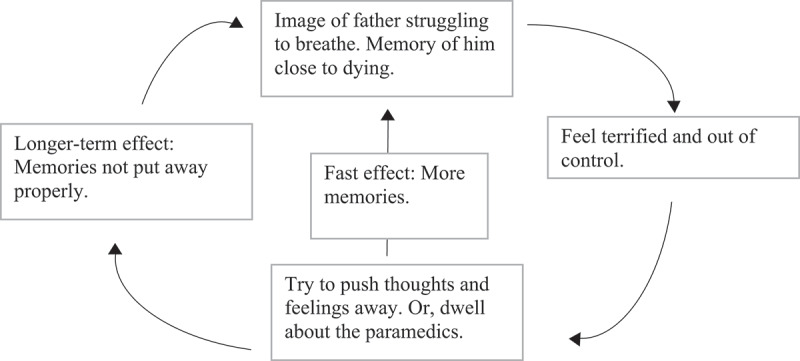
Maintenance cycle showing how the patient’s strategies for dealing with unwanted memories cause her symptoms to persist.

Figure 2 shows the maintenance cycle the therapist drew with the patient while sharing screen during their first video conferencing session after the diagnostic assessment. Kaja had discovered in the thought suppression experiment that trying to suppress an image made it pop into her mind.

### 1. 3. Reclaiming/rebuilding life assignments

Reclaiming or rebuilding life assignments help the patient to engage in enjoyable and/or meaningful activities they have stopped since the trauma or would like to work towards. The therapist begins planning these with the patient in the first session and every treatment session will include a discussion of progress as well as steps to build on this progress. This helps the patient to feel less stuck with the trauma, giving a sense of moving on with their life. The activities fit within life domains that are important to the patient, such as free time (e.g. interests and hobbies), work or study, home life, relationships and health (e.g. exercise and self-care). Some activities can be fully restarted immediately, while others need to be built up gradually.

Reclaiming life assignments serve many purposes. They instil hope, help to improve mood and support patients with steps towards reaching their goals. They can also help the patient access memories of their former selves when they were well and serve as a context for the trauma memory work. They help modify trauma-related negative appraisals about having permanently changed for the worse. Reclaiming life assignments can be planned as experiments to test beliefs about trauma sequelae, such as ‘I’ll never have energy again. If I try 10 minutes of exercise, I will be exhausted’, the outcome of which leads to experiential learning that can update appraisals and support behavioural change (in this example, that short bursts of exercise increase energy, which can then support the patient to incorporate exercise into their daily routine). Such assignments can be carried out together in session or set for homework. When planned as behavioural experiments, reclaiming life assignments model the approach that will be taken in therapy of identifying beliefs that may be maintaining symptoms, putting them to the test and gathering evidence to update them.

During social distancing and lockdown restrictions, many outdoor, work or social activities the therapist and patient would have planned are not possible for the patient to carry out in person. However, many can be adapted or achieved in an alternative way. When working remotely, the therapist and patient will consider together which activities are possible, which can be adapted, and those that are not possible and therefore need to go into the Blueprint (see section 2.6) as a list for future plans. Table 2 shows some examples of reclaiming life activities that patients can carry out despite restrictions during COVID-19. The choice depends on what would be in line with the patient’s goals and meaningful or enjoyable to them.

**Table 2.** Reclaiming life activities that can be carried out remotely.

Exercise	Finding your trainers again Going for a walk/run/cycle Health and fitness apps Taking the dog for a walk	Exercising at home using online videos, video game consoles or DVDs Yoga, Pilates Dancing
Self-care	‘Me time’ Relaxation activities Meditation/wellbeing apps Prayer/religious activities Buying yourself a treat Buying/making your favourite food	Taking a bath Getting back to your old beauty/grooming routine Trying a new style of clothes, hair or make-up Healthy eating Improving your sleep routine
Interests & Hobbies	Reading Creative writing, blogging Drawing, Arts and Crafts Learning a new skill/language (using the internet, books, or apps) Taking/organising photos Listening to or making music/playing an instrument Researching future holiday destinations	Cooking/baking (e.g. from your old recipes) DIY Gardening, growing seeds Crosswords/puzzles/quizzes Singing, joining an online choir Researching a topic of interest Watching TV or films that you used to enjoy
Social & Relationships	Planning a meal or activity with your family/partner/housemates/friends (in person, virtually, or both as required) Arranging to meet a friend or friends for virtual coffee/drink/dinner Catching up with work colleagues Checking in on neighbours/those who are vulnerable Playing a game online with a friend	Contacting someone you haven’t spoken to in a while (e.g. phone, email, messaging) Dating apps Online classes or discussion groups on social media Sending someone a card, letter or gift Activities with children (e.g. sports day, treasure hunt, make a movie, build something, board games) Organise an online quiz
Work	Researching job or volunteering opportunities Creating a pleasant workspace at home Online training courses or learning new skills to help to you improve/progress in your job role Updating your CV Starting/progressing/finishing a project or assignment	Evening classes, college, or university courses via distance learning Talking to your manager about how they can support you Seeking advice and help around money/benefits/employment Planning your work and career goals

For example, Kaja who developed PTSD following her father’s illness in a care home had given up going for long country walks. She had lost interest and believed it would make her more anxious since she wouldn’t have mobile reception to take an emergency call about her father. During the COVID pandemic, with her therapist, Kaja identified online exercise classes to try. When she was working out, she experimented with leaving her phone in another room. She discovered that she enjoyed the short classes and noticed that whilst focusing on the class, she worried much less about her father, which motivated her to try country walks where reception would come and go.

### 1. 4. Memory-focused techniques

#### 1. 4. 1. Updating trauma memories

CT-PTSD involves updating trauma memories to identify and modify trauma-related meanings that maintain the patient’s sense of current threat to their self-view or safety. A core component of this procedure is to help the patient engage with the moments in memory that carry the threatening meanings that still trouble the patient. During therapy, new less threatening meanings are discovered, linked to the memory, and helped to ‘sink in’ emotionally. The therapist’s aim is to facilitate an optimal level of engagement with the trauma memories so that patients can access the most distressing moments and their meanings while staying aware of the present and where they are at all times. We have mentioned above that this is harder for the therapist to manage over the phone than in face-to-face or video sessions, but is still possible with some adaptations, i.e. expressing empathy regularly through tone of voice and supportive statements, eliciting regular feedback from the patient and ratings of nowness and distress (for example, when a patient goes very quiet during a phone call), using reminders of the here and now to focus the patient on the present as needed. The choice of method to engage patients with the memory is also important here.

Two techniques are helpful for accessing the trauma memory and its meanings: imaginal reliving (Foa & Rothbaum, 1998) and/or narrative writing (Resick & Schnicke, 1993). In imaginal reliving, the therapist guides the patient to visualize the trauma and to describe events in the sequence in which they happened in an emotionally engaged way. Narrative writing involves writing out a detailed moment-by-moment account of the trauma and tends to be less emotional than reliving as the focus is on jointly producing a written account.

If the trauma is prolonged or the patient dissociates or lost consciousness during the trauma or the therapist is conducting CT-PTSD over the telephone, we recommend narrative writing as it allows the therapist more opportunities to pace the memory work and direct patients towards the memory content or away from it to the present situation as needed.

The updating memory procedure can be carried out effectively with patients remotely in a similar way as in face-to-face therapy. Table 3 summarises the essential practicalities for updating memories with adaptations for remote working. Video illustrations are also available on the COVID-19 page of a free therapist resources website (www.oxcadatresources.com).

**Table 3.** Essential practicalities for the in person and remote updating trauma memories procedure.

In Person & Remote Delivery		Purpose
Give rationale (e.g. to help put the trauma in the past; update the most distressing moments so they no longer feel like they’re happening now)		Increases understanding and engagement with the trauma-focused work.
Elicit and address concerns about reliving or narrative writing		Guides adaptations (e.g. conducting imaginal reliving for a part of the trauma in the first instance rather than the whole story) and informs behavioural experiments to test patient’s concerns, if necessary (e.g. testing predictions that the patient will not be able to stop crying, for example, if they talk through their trauma).
Agree where in the trauma story to start and stop. Generally, start before the main traumatic event and continue until the person felt safer, or the worst parts are over.		Provides clarity for the patient. Stopping reliving or narrative writing at a point where the patient felt safe ensures the patient is not left in moments of heightened distress and helps to demarcate the end of the traumatic event, wherever possible.
Allocate enough time to finish reliving or narrative writing.		This ensures you do not finish at the worst moment of the trauma associated with heightened distress, but rather have time to finish the trauma story at a point where the patient felt reasonably safe.
Be aware of avoidance		Spotting signs of avoidance (e.g. skipping over parts of the trauma) can help you to engage patients with their memory of the trauma in future reliving/narrative writing/updating and ensures that memory updating will be maximally effective for patients.
Identify worst moments (‘hot spots’)		Identifying the worst moments helps identify the worst meanings that are maintaining distress and symptoms.
Take ratings of distress and newness		These ratings show levels of distress and how much the memory seemed as though it was happening now rather than being in the past. When updating information is linked to worst moments, we would expect shifts in these ratings.
Identify updating information		This information will help to make the meanings less threatening and distressing. Information may emerge from knowledge about how the event unfolded (e.g. the outcome was better than expected) or from cognitive restructuring of meanings.
Include updates early		For outcomes that were better than expected (e.g. ‘I survived’; ‘I am still living with my family’) include such updating information in early reliving/narrative writing since this helps to reduce distress and increases hope and motivation.
Insert updates in narrative writing in a different colour		This helps to reinforce the updating information and helps to reduce distress.
Ask for feedback		Helps you to understand the patient’s experience and gives you information to make adaptations, if necessary.
Capture updates in a written flashcard or a photo		This helps patients to easily access updates. Flashcards and photos can be accessed on their phone.
Plan activity (e.g. a reclaiming life or self-care activity) the patient will do after the session		Rewards patients’ efforts, ensures they will move onto a cognitive or behavioural activity that is not trauma-focused, and may reduce opportunities for rumination.
**Adaptations for Remote Delivery**		
Check the patient has privacy		This helps to create a safe place for the patient to engage with the trauma memory.
Elicit any concerns the patient has about remote work		Provides the opportunity to address specific concerns and may likely increase the sense of safety with remote therapy.
For video conferencing sessions, ask the patient to show you their reminders of the here and now		Allows you to refer to specific reminders to bring the patient’s attention back to the here and now if they begin to dissociate.
For video conferencing sessions, routinely give patients the choice of reliving or writing a trauma narrative together		Increases the patient’s sense of control. If writing a trauma narrative, share screen. Patient or therapist can type the narrative. Use your voice and reminders of the here and now if the patient over-engages with the memory.
For telephone sessions, conduct narrative writing		Narrative writing is recommended for phone calls because we think it is important for the therapist to be able to see emotional reactions during reliving which is not possible during a phone call. The narrative can be written in session or for homework and emailed to you in advance of the call. To increase emotional engagement, especially when updating hot spots, ask the patient to read the narrative out loud or read it out to them if they prefer.

Updating trauma memories involves three steps:

- *Identify threatening personal meanings*. Imaginal reliving or narrative writing and discussion of the content of intrusive memories help the therapist and patient to identify the moments during the trauma associated with the most distress and sense of ‘nowness’ (‘hot spots’, Foa & Rothbaum, 1998). It is at these moments that the most important meanings can be identified. Screen sharing/exchanging the narrative via email helps ensure therapist and patient can work together collaboratively during narrative writing.
- *Identify updating information*. Updating information helps to make the meanings of the worst moments of the trauma (‘hot spots’) less threatening. This information may be garnered from what the patient knows now, such as that the outcome was better than expected (e.g. they survived; they are still living with their family), or it may become apparent when the patient recalls the trauma during imaginal reliving or narrative writing, such as remembering that the perpetrator had a weapon and this was the reason the patient chose to comply rather than fight back, or that the staff in the intensive care unit were trying to save the patient’s life rather than torture them (see Murray et al., 2020 for details). A range of cognitive restructuring techniques can be used to update appraisals linked to guilt, shame, anger, permanent change and an enduring sense of degradation and low self-worth. These include Socratic questioning, surveys, pie charts, behavioural experiments, imagery techniques, and positive data logs.
- *Link the new information to the relevant moment in memory*. Once the therapist and patient have discovered information that updates the patient’s meanings for a particular hot spot, the therapist guides the patient to hold the hot spot in mind whilst recalling or experientally taking in the new information. This can be done verbally (e.g. ‘I now know …’) whilst bringing the hot spot to mind in reliving or when reading through the hot spot and the updates (highlighted in a different colour) in the narrative. Where possible, the new information can be strengthened or demonstrated experientially or with imagery that shows the worst fears did not happen (e.g. picturing a loved one who died at peace in one’s mind or looking at a photo of an occasion with a loved one since the trauma to show that the patient survived), with movement (e.g. moving around freely to show they are no longer trapped), or with incompatible sensations (e.g. touching a healed body part). Hot spots are updated one-by-one as early as possible, which can be in the same session that the hot spot was identified if the updates are straightforward. As updating usually leads to a large shift in emotion and improvements in intrusions and sleep, the early updates help with creating hope and motivation for treatment. When updating is done with narrative writing, we recommend that therapist and patient screen share and insert the new, updating information into the narrative using a different colour and then read out both together.

The order of interventions in CT-PTSD is flexible and adapted to the patient’s symptoms. As in face-to-face therapy, for patients with a history of dissociation in response to trauma reminders, the therapist and patient will work on managing dissociation, identifying triggers for dissociation and introducing trigger discrimination (described below) and reminders of the here and now before reliving or narrating the details of the trauma. For patients who are very ashamed about the trauma or experienced mental defeat, initial cognitive restructuring such as a survey about what other people think about the patient’s reactions and the perpetrators can also be helpful to do first.

When patients have experienced multiple traumatic events, it can be useful to draw a timeline of life events prior to reliving or narrative writing. This is especially the case if the person has re-experiencing symptoms associated with many events. The timeline often starts from birth and extends to the patient’s current age or it can cover the period of time during which the patient had exposure to multiple trauma. The timeline may also include the future to better consider the patient’s goals and future hopes. The patient indicates the age at which traumatic as well as positive life events occurred on the timeline. The therapist will also ask the patient to indicate which traumas are associated with re-experiencing symptoms in the present to guide which trauma to first work on with the updating memories procedure.

#### 1. 4. 2. Then vs Now trigger discrimination

Then vs Now trigger discrimination is used in CT-PTSD to reduce trauma memories being triggered. There are many stimuli that patients encounter in everyday life that can have some resemblance to the circumstances present in their trauma and can act as triggers. As well as obvious reminders (e.g. such as people who are same height and similar visual appearance as a perpetrator), triggers also may be more subtle sensory features (e.g. such as shade of colour, specific sounds, smells, tastes, and body position or movement). Such triggers can be hard for patients to spot initially, requiring joint detective work by therapist and patient. Sometimes when trauma memories are triggered, patients don’t remember the event but only re-experience the same strong emotions (intense fear, anger, sadness, shame) or body sensations (e.g. pain, nausea).

Then vs Now trigger discrimination aims to break the link between the triggers for intrusive memories in the present and the past trauma. The therapist and patient work together to spot triggers that reactivate the patient’s trauma memory, usually aided by keeping a diary of when and where intrusions occur. They then work together to break the link that has formed between the memory then and the triggers now by shifting the patient’s attention to all the ways the trigger is different to the memory. Therapist and patient often start by selecting a trigger that has reactivated the patient’s trauma memory in their everyday life, and drawing out similarities and differences before practising focusing the patient’s attention on the differences with a trigger in the session. It can be useful to intentionally enhance the perceptual differences by moving about, manipulating the volume of an audiofile etc.

*For example, Kaja reported that hearing ambulance sirens while she was talking to her father on the phone had triggered an intrusive memory of her father struggling to breathe in the care home before the paramedics arrived. The therapist helped Kaja to spot similarities and differences between the memory then and the trigger now. Table 4 shows the Then vs Now table they drew up together before they practised Then vs Now discrimination with an audio file of ambulance sirens, which the therapist emailed to Kaja during their session*.

In Kaja’s example, the trigger (sound of a siren) elicited a memory that included a visual detail of the traumatic event (seeing her father struggle to breathe). However, sometimes a trigger (household objects such as towels, books, etc. that are the same colour as PPE worn by staff in a COVID-19 ICU ward) may only elicit the emotional reaction during a traumatic event (the terror and loneliness when a recovered patient thought he/she might die) without the sensory source details of the memory (being in the intensive care unit). This type of triggering can be more difficult to spot, but once it has been spotted it is dealt with in the same way, by presenting the trigger stimuli and focusing on the differences in the safe here and now.

When working remotely with a video conferencing facility, the therapist and patient can share screen to work on the Then vs Now table together. For telephone sessions, if possible, the therapist will email a Word version of the table to the patient that they then complete together. For remote working, it is necessary to source possible triggers (images and audio files) before the session. The therapist will email or send via messaging relevant audio and image files before or during the session or afterwards to practise with for homework. They will then work on the trigger discrimination with the patient via screenshare in videoconferencing or by talking on the phone while the patient looks at/listens to the triggers. If the therapy is being conducted on the telephone, the therapist and patient will consider what triggers (such as objects/photos) the patient may have in their home and will work together with these.

#### 1. 4. 3. Site visit

A visit to the site of the trauma is a core component of CT-PTSD. Site visits help the patient to experience that the trauma is over; they may lead to discovering new information to update meanings; they can help to re-build the trauma memory; and test a belief or concern the patient has, such as that the patient will lose control and be unable to cope (Murray, Merritt, & Grey, 2015).

Carrying out site visits remotely is possible and acceptable to patients. If it is safe and social distancing restrictions allow for the patient to return to the site of the trauma, the therapist and patient plan the visit in advance. The patient can visit the site alone or with a supportive family member or friend. The therapist can speak to the patient on the phone while they are there to support them and guide them to notice differences between the site at the time of the trauma and how it is now and to discover further evidence that is relevant for their appraisals.

For example, Kaja did a remote site visit (via her laptop) to the care home with her therapist on the telephone who guided her to spot what was different about the care home today than the time of her father’s acute illness. Kaja spotted that flowers were blooming outside the care home whereas when her father was ill, the flower beds were bare. The therapist also asked Kaja to take a screen shot of her video conferencing calls with her father. In one of them, her father’s carer had come into his room. Kaja spotted that this was a new carer who made his tea and shared his interest in politics. She also spotted that her father had put up a framed screen shot of the two of them talking via videoconferencing taken after the trauma. These differences helped Kaja to experience that the trauma really was over.

If it is not possible for the patient to visit the site in person, visiting the site virtually via Google Street View is a good alternative. The therapist and patient can virtually access the site together in a telephone or video conferencing session. If video conferencing, the therapist or patient can share screen so they can look at the site together. They may also walk the route of the trauma virtually, paying close attention to differences between then and now. Google Earth can be used for the parts of the world where Google Street View is unavailable and allows the therapist and patient to view the site from above and zoom in. For traumas that happened inside sites, such as hospitals or other buildings, that cannot be accessed via Street View, the therapist may be able to source images of the outside, and sometimes interior, of the building and access virtual tours of similar buildings. Finally, Google Street View often shows images of the site taken at different dates, which help to show how the site has changed over time and can be used to reinforce the message that the trauma is in the past. Taking photos and reviewing them later is another useful way of consolidating the learning from the site visit. If the patient does visit the site, they can take a ‘selfie’ or, for virtual site visits, screenshots of important images can be taken by the patient, or by the therapist and emailed to the patient.

When conducting the virtual site visit remotely in session, and sharing screen, the patient’s view of the site will be small if the therapist is sharing screen and larger if the patient is sharing screen. The therapist will need to check during the session whether or not the trauma memory has been triggered, and if so, to use then vs now to help focus the patient on differences between the trigger and the memory. If the patient is sharing screen, then the therapist can encourage them to slow down if they are rushing through the virtual site visit and ensure they are focused on the site rather than avoiding aspects of it.

### 1.5. Modifying problematic appraisals

When working remotely, the therapist can use the same tools as in face-to-face therapy to work with meanings linked to common cognitive themes associated with PTSD, such as cognitions leading to shame, guilt, anger, loss, permanent change, overgeneralised sense of danger or persisting sense of degradation and low self-worth. This cognitive restructuring will involve a combination of psychoeducation, Socratic questioning, surveys, pie charts, behavioural experiments and/or imagery techniques and other cognitive therapy techniques.

Working with appraisals of shame, for example, often involves creating a survey with patients to elicit the compassionate views of other people and the key message that other people would accept and understand rather than reject or judge the patient if they knew what happened. Behavioural experiments help the patient to further test their predictions about how other people would respond if they shared their experience.

Working with appraisals of guilt includes creating a responsibility pie chart to help the patient consider all contributing factors for the trauma or the behaviour that they feel guilty about. The aim is to reduce the belief that they are responsible for the trauma or that it means something bad about themselves. The therapist will also guide the patient to focus on what they did during and since the trauma that has been helpful.

For example, Kaja thought that she should have challenged the paramedics and insisted that they take her father to hospital for oxygen therapy to help his breathing, and because of this, she believed that she was 100% responsible for his suffering. In remote therapy, using shared screen, Kaja and the therapist drew out a responsibility pie chart in which they looked at all the reasons why her father had suffered. He was elderly; he had had a severe chest infection during the peak of the COVID pandemic, which may have affected clinical decision-making; the paramedics who were called to the care home were blunt when they told her his prognosis and this put her in a state of shock, which made it difficult to think clearly and insist on treatment. In considering all these possible reasons contributing to her father’s suffering, Kaja re-rated her own responsibility as 10%. She also discovered that had the paramedics taken him to hospital, he may have died as there would have been patients in hospital with COVID. She concluded that not challenging the paramedics may have saved his life since he was able to avoid contact with many ill patients.

When working with appraisals linked to anger, psychoeducation about the reasons for other people’s behaviour may be important (e.g. why the police take DNA samples of victims of violence or why A&E staff do not explain procedures during emergencies) as well as considering the advantages and disadvantages of holding onto anger. It can be helpful for the patient to write an ‘anger letter’, which they do not send, to the person they are angry with clearly spelling out why they are angry and what they want the person who harmed them to know. The therapist may also conduct a survey to gather information to normalise how the patient is feeling and support them to let go of anger.

With themes of loss, the therapist will help the patient to remember the person as a whole and what they brought to the patient’s life; what they valued about them. The therapist may gradually guide the client to discover what has not been lost, and will often use imagery to help update worst meanings. For example, if a patient has lost a loved one through trauma, the therapist may guide the patient to picture in imagery their loved one at peace or picture an image that captures the meaning of their loved one which lives on (e.g. picturing sunshine to capture their loved one’s warm-heartedness and positivity). When there is loss related to COVID or other death trauma associated with suffering, such as death by cancer or suicide, the therapist will guide the client to consider as updating information that the loved one is no longer suffering. The therapist will guide the patient to link this information to the relevant worst moment in the trauma in imaginal reliving or in narrative writing.

For themes of unacceptable change to their body, the therapist will guide the patient to consider ways to rebuild their life and find meaningful changes to their activities. The therapist may guide the patient to discover what has not been lost, use Then vs Now discrimination when intrusive memories trigger feelings of vulnerability as well as videofeedback to help update the patient’s perceptions of how they fear they look with objective information of how they look now. Finally, for overgeneralised sense of danger, the therapist will use Socratic questioning to guide the patient to discover that objective risk has not changed. Therapist and patient will calculate estimates of objective risk from the patient’s past experience or statistics and compare them to the patient’s felt sense of risk and refer to questionnaires, such as the Safety Behaviours Questionnaire, to identify safety behaviours that may be maintaining the patient’s sense of threat and plan experiments for the patient to drop these. During the COVID-19 pandemic, overgeneralised sense of danger may extend to a fear of catching the virus and dying. This may be particularly true for patients who have experienced hospital-related trauma, such as a traumatic childbirth. The therapist would guide the patient to discover that objective risk of dying has not changed substantially, drawing on readily available evidence. For example, on 24 May 2020, the BBC published data, demonstrating that the risk of dying from coronavirus mirrors the chances of dying from any cause within a given year and that this risk varies depending on age  (BBC, 2020). For example, for a 40 year old, the risk of dying from coronavirus is around 0.01%, the same as the risk of dying from any cause over their next 12 months. Thus, the actual risk of death for a 40 year old from any cause, including coronavirus, is 0.02%. This risk slowly rises over time, reaching a risk of death by coronavirus of 10% for someone who is 90 years old or 20% for any cause, including coronavirus, at that age. The therapist would help the client to discover that their feeling of risk is driven by their trauma memory, which may be activated in response to triggers, as well as the behaviours they may engage in to feel safe, such as checking for danger, which keeps them focused on threat. The therapist would help the patient to discriminate between triggers in the present (e.g. face masks) and their past trauma. The therapist would also guide the patient to experiment with dropping safety behaviours, such as excessive checking for symptoms or news reports of coronavirus cases. The therapist may also guide the patient to remind themself that helpful measures are in place to reduce the spread of the virus and these appear to have been successful in many countries. If the patient has had hospitalisation in intensive care for COVID and has subsequently developed PTSD, the core interventions described in this paper can be used to treat their PTSD. Please see Murray et al. (2020) for details.

If using a videoconferencing facility, the therapist can share screen to look at questionnaires, plan experiments, plan and review surveys, draw pie charts, calculate risk with sequential probabilities and read anger letters with the patient. If working over the telephone, the therapist can email questionnaires, behavioural experiment record sheets, completed surveys and blank or completed pie charts to the patient before or during the session, which the patient can access on their home computer at the same time as they have their telephone call.

### 1. 6. Dropping unhelpful maintaining behaviours and cognitive strategies

Often the first step in addressing maintaining behaviours and cognitive strategies is to elicit their advantages and disadvantages and then carry out a behavioural experiment to test the effect of increasing and decreasing the strategies. During lockdown and social distancing, the therapist and patient are unlikely to be able to access outdoor situations together, such as crowds or busy roads or other situations directly relevant to the patient’s trauma. However, the patient can carry out an experiment at home to elicit the general principle: that excessive checking increases the sense of threat, and then generalise the learning to their trauma. They may then apply the learning to a situation more directly relevant to their trauma, such as busy road or a supermarket where there will be people. If the therapist is speaking to the patient during the experiment, it will be important to check the patient is dropping their safety behaviours. The therapist can also guide the patient to use then vs now if the trauma memory is triggered.

For example, since the trauma, Kaja rang the care home twice a day to ask them to check on her father’s breathing and emailed in between phone calls. With her therapist, Kaja discovered some of the disadvantages to her constant checking. She thought it was likely bothering the staff who had many patients to care for, it took up time and it made her question whether they had checked ‘properly,’ which made her feel anxious about his health. Having spotted disadvantages, Kaja was motivated to experiment with increasing and decreasing her checking to see if anything bad happened and how this would affect her anxiety. First, Kaja’s therapist guided her to test the principle that excessive checking increases the sense of risk by going into her kitchen and looking for danger. For 30 seconds, she looked for sharp objects, sharp edges, checked whether the oven was off and whether the knives had been put away properly. She rated her anxiety as 90%. Kaja then dropped her checking for 30 seconds, making a cup of tea with her full attention. She rated her anxiety as 10%, concluding that checking for danger had made her feel more anxious. The therapist guided Kaja to apply this learning to her father’s health. Kaja experimented with calling the care home once a day and speaking to her dad rather than asking staff to check his breathing. She also stopped following up with emails. She discovered that she had more time for reclaiming life activities and that she felt a lot less anxious than when she was constantly checking on her father.

In working with rumination, the therapist will guide the patient to discover the effects of rumination on mood, the types of thoughts that characterise rumination and whether or not such thoughts lead to answers or a productive plan of action. A metaphor of a broken-down car may be used to illustrate the unproductive nature of rumination. The patient is asked to imagine their car has broken down and that in one situation, they respond with thoughts, such as ‘Why is this happening to me?’ and in another, with thoughts, such as ‘How can I resolve this problem?’ This helps to illustrate the unproductive nature of ruminative ‘Why?’ thoughts and the patient is guided to apply this learning to their trauma. This can be done over the telephone or by videoconference. It can be helpful for patients to label their rumination when they notice themselves ruminating, e.g. ‘I’m dwelling again’, ‘there goes my circular thinking’. The therapist will then work with the patient to develop a plan to disengage from rumination, putting in place strategies that help them to move onto other activities that get them out of their head and into the world when they first spot signs of dwelling.

### 1. 7. Blueprint

The blueprint is a relapse prevention plan that summarises key learning the patient has gained from treatment, steps to build on this learning and how to approach anniversaries of the trauma and potential future setbacks. The blueprint includes six questions, which are shown in Table 5.

**Table 4.** Then vs Now trigger discrimination table developed for one of the patient’s triggers (ambulance siren).

	THEN	NOW
Similarities	Ambulance on road	Ambulance on road
	Sirens	Sirens
Differences	9 am in the morning	7 pm at night
	Father can’t breathe	Father is breathing
	My father could barely speak	I can hear my dad chatting on the phone
	My father is uncomfortable and in pain	My father is comfortable
	Hearing the ambulance suggested danger, something terrible might happen to my dad	Hearing the ambulance means the paramedics may be able to help someone else. My dad is safe.

**Table 5.** Questions asked in the blueprint.

How did my problems develop? What kept my problems going? What did I learn during treatment that helped? What were my most unhelpful thoughts? What are the helpful alternatives/updated thoughts? How will I deal with any setbacks in the future?

Prompts help to guide patients’ responses. The blueprint can be emailed to the patient. Typically the patient will complete the blueprint as a homework assignment, email it back to the therapist, and in a subsequent session, the therapist reviews it with the patient over the telephone or by sharing screen over video conferencing, agreeing with the patient additional content to add where appropriate.

## 2. Discussion

CT-PTSD is a highly effective treatment for PTSD that in our experience can be delivered remotely over video conferencing facility or the telephone, using the same treatment components as in face-to-face delivery and in their typical order, with some adjustments. A trial is currently underway evaluating therapist-supported internet-delivered cognitive therapy for PTSD (Ehlers et al., 2020b), a remote version of CT-PTSD that is acceptable to patients, includes modules for the patient to complete and shows promising preliminary results (Wild et al., 2016). However, this and other web-based versions of trauma-focused CBT are not yet routinely available in clinical services in the UK. In the US, clinical trials show that remote delivery of trauma-focused PTSD treatments, such as cognitive processing therapy or prolonged exposure therapy, are as good as in-person delivery of such treatments (i.e. Acierno et al., 2017; Morland et al., 2015) although it is unclear if these treatments are routinely available. In this paper, we have described how to adapt the core interventions of CT-PTSD to deliver them over video conferencing facilities or the telephone on the basis of our experience with treating patients with a wide range of traumas remotely in this trial. However, we acknowledge we do not have sufficient data to confirm that the effect size of treatment remains exactly the same and some patients may find it harder to engage with therapy remotely. However, remote therapy makes treatment available to patients who would find it impossible/difficult to attend face-to-face sessions such as people with health problems that affect mobility or parents with young babies. During the COVID-19 pandemic, social distancing policies are likely to evolve according to the rise or decline of COVID cases, which will influence how psychological therapies can be delivered. It is possible that for some patients, they may begin their therapy remotely then have opportunity to attend face-to-face sessions. It will be up to the patient to decide if they wish to continue their remote therapy or to revert to face-to-face sessions if given the opportunity. The therapist can guide decision-making by helping the client to consider the advantages and disadvantages of remote versus in-person therapy.

We also acknowledge that remote therapy can pose challenges for therapists and are mindful that many therapists will be delivering the therapy out of their own homes. We encourage therapists to extend the compassion they extend to patients to themselves, to take breaks after speaking to patients and to keep a clear distinction between work and home life. It can help to put remote therapy tools (laptop, work mobile phone, image or audio files of triggers that have been sourced for patients) away after calls and preferably in a different room. Hearing about a patient’s trauma at home can be especially distressing if there are parallels to the therapist’s private life, which may become more common with COVID-19 related trauma and bereavement affecting large proportions of the population in many countries. Therapists may find it helpful to use Then vs Now discrimination themselves at home when they are winding down, if needed, spotting what is different about their home environment and situation to the patient’s trauma. It may also be helpful to have a balance between PTSD and other cases and to seek support from managers and supervisors as required.

In conclusion, CT-PTSD is a highly acceptable and effective treatment that can be offered to patients with PTSD during the COVID pandemic remotely with a few practical adaptations. These adaptations may also be applicable to other forms of trauma-focused CBT.

## References

[cit0001] Acierno, R., Knapp, R., Tuerk, P., Gilmore, A. K., Lejuez, C., Ruggiero, K., … Foa, E. B. (2017). A non-inferiority trial of prolonged exposure for posttraumatic stress disorder: In person versus home-based telehealth. *Behaviour Research and Therapy*, 89, 57–15. 27894058 10.1016/j.brat.2016.11.009 PMC5222772

[cit0002] American Psychological Association. (2017). Clinical practice guideline for the treatment of posttraumatic stress disorder (PTSD) in adults. Retrieved from http://www.apa.org/ptsd-guideline/ptsd.pdf

[cit0003] Ashwick, R., Turgoose, D., & Murphy, D. (2019). Exploring the acceptability of delivering cognitive processing therapy (CPT) to UK veterans with PTSD over Skype: A qualitative study. *European Journal of Psychotraumatology*, 10, 1. 10.1080/20008198.2019.1573128 PMC6366402 30774784

[cit0004] BBC. (2020, 5 24). Coronavirus: How scared should we be? Retrieved from https://www.bbc.co.uk/news/health-52758024

[cit0005] Clohessy, S., & Ehlers, A. (1999). PTSD symptoms, response to intrusive memories, and coping in ambulance service workers. *British Journal of Clinical Psychology*, 38, 251–265. 10.1348/014466599162836 10532147

[cit0006] Duffy, M., Gillespie, K., & Clark, D. M. (2007). Post-traumatic stress disorder in the context of terrorism and other civil conflict in Northern Ireland: Randomised controlled trial. *British Medical Journal*, 334, 1147–1150. 17495988 10.1136/bmj.39021.846852.BE PMC1885307

[cit0007] DuHamel, K. N., Mosher, C. E., Winkel, G., Labay, L. E., Rini, C., Meschian, Y. M., … Redd, W. H. (2010). Randomized clinical trial of telephone-administered cognitive-behavioral therapy to reduce post-traumatic stress disorder and distress symptoms after hematopoietic stem-cell transplantation. *Journal of Clinical Oncology: Official Journal of the American Society of Clinical Oncology*, 28(23), 3754–3761. 20625129 10.1200/JCO.2009.26.8722 PMC2917309

[cit0008] Dunmore, E., Clark, D. M., & Ehlers, A. (2001). A prospective study of the role of cognitive factors in persistent posttraumatic stress disorder after physical or sexual assault. *Behaviour Research and Therapy*, 39(9), 1063–1084. 11520012 10.1016/s0005-7967(00)00088-7

[cit0009] Ehlers, A. (2013). Trauma-focused cognitive behavior therapy for posttraumatic stress disorder and acute stress disorder. In G. Simos & S. G. Hofmann (Eds.), *Textbook of CBT for anxiety disorders* (pp. 161–189). New York: Wiley.

[cit0010] Ehlers, A., & Clark, D. M. (2000). A cognitive model of posttraumatic stress Disorder. *Behaviour Research and Therapy*, 38(4), 319–345. 10761279 10.1016/s0005-7967(99)00123-0

[cit0011] Ehlers, A., Clark, D. M., Hackmann, A., McManus, F., & Fennell, M. (2005). Cognitive therapy for post-traumatic stress disorder: Development and evaluation. *Behaviour Research and Therapy*, 43(4), 413–431. 15701354 10.1016/j.brat.2004.03.006

[cit0012] Ehlers, A., Clark, D. M., Hackmann, A., McManus, F., Fennell, M., Herbert, C., & Mayou. (2003). A randomized controlled trial of cognitive therapy, a self-help booklet, and repeated assessments as early interventions for posttraumatic stress disorder. *Archives of General Psychiatry*, 60(10), 1024–1032. 14557148 10.1001/archpsyc.60.10.1024

[cit0013] Ehlers, A., Grey, N., Warnock-Parkes, E., Wild, J., Stott, R., & Clark, D. M. (2020a). Effectiveness of cognitive therapy in routine clinical care: Second phase implementation. Submitted for publication.

[cit0014] Ehlers, A., Grey, N., Wild, J., Stott, R., Liness, S., Handley, R., … Clark, D. M. (2013). Implementation of cognitive therapy in routine clinical care: Effectiveness and moderators of outcome in a consecutive sample. *Behaviour Research and Therapy*, 51(11), 742–752. 24076408 10.1016/j.brat.2013.08.006 PMC3897916

[cit0015] Ehlers, A., Hackmann, A., Grey, N., Wild, J., Liness, S., Albert, I., … Clark, D. M. (2014). A randomized controlled trial of 7-day intensive and standard weekly cognitive therapy for PTSD and emotion-focused supportive therapy. *American Journal of Psychiatry*, 171(3), 294–304. 10.1176/appi.ajp.2013.13040552 PMC4082238 24480899

[cit0016] Ehlers, A., Wild, J., Warnock-Parkes, E., Grey, N., Murray, H., Kerr, A., & Clark, D. M. (2020b). A randomised controlled trial of therapist-assisted online psychological therapies for posttraumatic stress disorder (STOP-PTSD): Trial protocol. *Trials*, 21, 355. 32326954 10.1186/s13063-020-4176-8 PMC7181498

[cit0017] Ehlers, A., Wild, J., Warnock-Parkes, E., Stott, R., Grey, N., & Clark, D. M. (2020c). Efficient use of therapist time in the treatment of posttraumatic stress disorder: A randomized clinical trial of brief self-study assisted and standard weekly cognitive therapy for PTSD. Submitted for publication.

[cit0018] Foa, E. B., Ehlers, A., Clark, D. M., Tolin, D., & Orsillo, S. (1999). The post-traumatic cognitions inventory (PTCI). Development and validation. *Psychological Assessment*, 11, 303–314.

[cit0019] Foa, E. B., & Rothbaum, B. O. (1998). *Treating the trauma of rape. Cognitive-behavior therapy for PTSD*. New York, NY: Guilford Press.

[cit0020] International Society of Traumatic Stress Studies. (2019). Posttraumatic stress disorder prevention and treatment guidelines. Retrieved from http://www.istss.org/getattachment/Treating-Trauma/New-ISTSS-Prevention-and-Treatment-Guidelines/ISTSS_PreventionTreatmentGuidelines_FNL.pdf.aspx 10.1002/jts.22421 31283056

[cit0021] Kleim, B., Grey, N., Hackmann, A., Nussbeck, F., Wild, J., Stott, R., … Ehlers, A. (2013). Cognitive change predicts symptom reduction with cognitive therapy for posttraumatic stress disorder. *Journal of Consulting and Clinical Psychology*, 81, 383–393. 23276122 10.1037/a0031290 PMC3665307

[cit0022] Knaevelsrud, C., & Maercker, A. (2007). Internet-based treatment for PTSD reduces distress and facilitates the development of a strong therapeutic alliance: A randomized controlled clinical trial. *BMC Psychiatry*, 7(1), 13. 17442125 10.1186/1471-244X-7-13 PMC1885249

[cit0023] Kroenke, K., Spitzer, R., & Williams, J. B. (2001). The PHQ-9: Validity of a brief depression severity measure. *Journal of General Internal Medicine*, 16, 603–613. 10.1046/j.1525-1497.2001.016009606.x PMC1495268 11556941

[cit0024] Kuester, A., Niemeyer, H., & Knaevelsrud, C. (2016). Internet-based interventions for posttraumatic stress: A meta-analysis of randomized controlled trials. *Clinical Psychology Review*, 43, 1–16. 26655959 10.1016/j.cpr.2015.11.004

[cit0025] Kuhn, E., Kanuri, N., Hoffman, J. E., Garvert, D. W., Ruzek, J. I., & Taylor, C. B. (2017). A randomized controlled trial of a smartphone app for posttraumatic stress disorder symptoms. *Journal of Consulting and Clinical Psychology*, 85(3), 267‐273. 10.1037/ccp0000163 28221061

[cit0026] Lange, A., Van de Ven, J. P., & Schrieken, B. (2003). Interapy: Treatment of post-traumatic stress via the internet. *Cognitive Behaviour Therapy*, 32(3), 110–124. 16291543 10.1080/16506070302317

[cit0027] Lewis, C., Roberts, N. P., Bethell, A., Robertson, L., & Bisson, J. I. (2018). Internet‐based cognitive and behavioural therapies for post‐traumatic stress disorder (PTSD) in adults. *Cochrane Database of Systematic Reviews*, 12. Art. No.: CD011710. doi:10.1002/14651858.CD011710.pub2 PMC6516951 30550643

[cit0028] Litz, B. T., Engel, C. C., Bryant, R. A., & Papa, A. (2007). A randomized, controlled proof-of-concept trial of an Internet-based, therapist-assisted self-management treatment for posttraumatic stress disorder. *American Journal of Psychiatry*, 164(11), 1676–1684. 10.1176/appi.ajp.2007.06122057 17974932

[cit0029] Luxton, D. D., O'Brien, K., Pruitt, L. D., Johnson, K., & Kramer, G. (2014). Suicide risk management during clinical telepractice. *The International Journal of Psychiatry in Medicine,* *48*, 19–31. doi:10.2190/PM.48.1.c 25354924

[cit0030] Meiser-Stedman, R., Smith, P., McKinnon, A., Dixon, C., Trickey, D., Ehlers, A., … Dalgleish, T. (2017). Cognitive therapy versus wait list as an early intervention for PTSD in children and adolescents: A randomized controlled trial. *Journal of Child Psychology and Psychiatry*, 58(5), 623–633. 27976374 10.1111/jcpp.12673 PMC5362068

[cit0031] Michael, T., Ehlers, A., Halligan, S., & Clark, D. M. (2005). Unwanted memories of assault: What intrusion characteristics predict PTSD? *Behaviour Research and Therapy*, 43, 613–628. 15865916 10.1016/j.brat.2004.04.006

[cit0032] Morland, L. A., Mackintosh, M. A., Rosen, C. S., Willis, E., Resick, P., Chard, K., & Frueh, B. C. (2015). Telemedicine versus in‐person delivery of cognitive processing therapy for women with posttraumatic stress disorder: A randomized noninferiority trial. *Depression and Anxiety*, 32, 811–820. 26243685 10.1002/da.22397

[cit0033] Murray, H., Grey, N., Wild, J., Warnock-Parkes, E., Kerr, A., Clark, D. M., & Ehlers, A. (2020). Cognitive therapy for post-traumatic stress disorder following critical illness and intensive care unit admission. *The Cognitive Behaviour Therapist*. doi:10.1017/S1754470X2000015X PMC7251252 34191936

[cit0034] Murray, H., Merritt, C., & Grey, N. (2015). Returning to the scene of the trauma in PTSD treatment–why, how and when? *The Cognitive Behaviour Therapist*, 8, E28. doi:10.1017/S1754470X15000677

[cit0035] National Institute of Health and Clinical Excellence (2018). Posttraumatic stress disorder. Retrieved from www.nice.org.uk/guidance/ng116

[cit0036] Olff, M., Amstadter, A., Birkeland, C., Bui, M. S., Cloitre, E., Ehlers, M., … Thoresen, S. (2019). A decennial review of psychotraumatology: What did we learn and where are we going? *European Journal of Psychotraumatology*, 10, 1. 10.1080/20008198.2019.1672948 PMC6924542 31897268

[cit0037] Price, M., Van Stolk-Cooke, K., Legrand, A. C., Brier, Z. M. F., Ward, H. L., Connor, J. P., … Skalka, C. (2018). Implementing assessments via mobile during the acute posttrauma period: Feasibility, acceptability and strategies to improve response rates. *European Journal of Psychotraumatology*, 9(sup1). doi:10.1080/20008198.2018.1500822 PMC6070964 30083303

[cit0038] Resick, P. A., & Schnicke, M. K. (1993). *Cognitive processing therapy for rape victims*. Newbury Park, CA: Sage. 10.1037//0022-006x.60.5.748 1401390

[cit0039] Sander, L. B., Schorndanner, J., Terhorst, Y., Spanhel, K., Pryss, R., Baumeister, H., & Messner, E. M. (2020). ‘Help for trauma from the app stores?’ A systematic review and standardised rating of apps for post-traumatic stress disorder (PTSD). *European Journal of Psychotraumatology*, 11(1). doi:10.1080/20008198.2019.1701788 PMC6968629 32002136

[cit0040] Simon, N., McGillivray, L., Roberts, N. P., Barawi, K., Lewis, C. E., & Bisson, J. I. (2019). Acceptability of internet-based cognitive behavioural therapy (i-CBT) for post-traumatic stress disorder (PTSD): A systematic review. *European Journal of Psychotraumatology*, 10, 1. 10.1080/20008198.2019.1646092 PMC6719262 31497259

[cit0041] Smith, P., Yule, W., Perrin, S., Tranah, T., Dalgleish, T. I. M., & Clark, D. M. (2007). Cognitive-behavioral therapy for PTSD in children and adolescents: A preliminary randomized controlled trial. *Journal of the American Academy of Child & Adolescent Psychiatry*, 46(8), 1051–1106. 17667483 10.1097/CHI.0b013e318067e288

[cit0042] van der Meer, C. A. I., Bakker, A., Zuiden, M. V., Lok, A., & Olff, M. (2020). Help in hand after traumatic events: A randomized controlled trial in health care professionals on the efficacy, usability, and user satisfaction of a self-help app to reduce trauma-related symptoms. *European Journal of Psychotraumatology*, 11, 1. 10.1080/20008198.2020.1717155 PMC7144205 32284818

[cit0043] Weathers, F. W., Litz, B. T., Keane, T. M., Palmieri, P. A., Marx, B. P., & Schnurr, P. P. (2013). The PTSD checklist for DSM-5 (PCL-5). *Scale available from the National Center for PTSD at www. ptsd.va.gov, 10*.

[cit0044] Weiss, D. S. (2007). The impact of event scale-revised. In J. P. Wilson & T. M. Keane (Eds.), *Assessing psychological trauma and PTSD: A practitioner’s handbook*. New York: Guilford Press.

[cit0045] Wild, J., Warnock-Parkes, E., Grey, N., Stott, R., Wiedemann, M., Canvin, L., … Ehlers, A. (2016). Internet-delivered cognitive therapy for PTSD: A development pilot series (pp. 399–411). *European Journal of Psychotraumatology*, 7(1), 31019. 27837579 10.3402/ejpt.v7.31019 PMC5106866

